# Variable Distribution of DOCK-D Proteins between Cytosol and Nucleoplasm in Cell Lines, Effect of Interleukin-4 on DOCK10 in B-Cell Lymphoid Neoplasms, and Validation of a New DOCK10 Antiserum for Immunofluorescence Studies

**DOI:** 10.3390/antib10030033

**Published:** 2021-08-20

**Authors:** Natalia Ruiz-Lafuente, Alfredo Minguela, Jose M. Moraleda, Manuel Muro, Antonio Parrado

**Affiliations:** 1 Department of Immunology, University Clinical Hospital Virgen de la Arrixaca-Biomedical Research Institute of Murcia (IMIB-Arrixaca), 30120 Murcia, Spain; natalia-ruizlaf@hotmail.com (N.R.-L.); alfredo.minguela@carm.es (A.M.); manuel.muro@carm.es (M.M.); 2 Department of Hematology, University Clinical Hospital Virgen de la Arrixaca-Biomedical Research Institute of Murcia (IMIB-Arrixaca), 30120 Murcia, Spain; jmoraled@um.es

**Keywords:** DOCK10, DOCK9, DOCK11, subcellular localization, B-cell lymphoid neoplasms, interleukin-4 (IL-4), membrane ruffles, filopodia

## Abstract

Dedicator-of-cytokinesis (DOCK), a family of guanine-nucleotide exchange factors (GEFs), comprises four subfamilies, named from A to D. DOCK-D comprises DOCK9, DOCK10, and DOCK11. The GEF activity involves translocation from the cytoplasm to the plasma membrane (PM), as assessed by the transfection of tagged proteins. However, the cellular localization of endogenous DOCK proteins is poorly understood. In this paper, to gain a better understanding of the role of the DOCK-D proteins, we studied their distribution between cytosol and nucleoplasm in 11 cell lines. DOCK-D proteins were distributed with variable cytosolic or nuclear predominance, although the latter was common for DOCK9 and DOCK11. These results suggest that the DOCK-D proteins may perform new nuclear functions, which remain to be discovered. Furthermore, we found that DOCK10 levels are increased by interleukin-4 (IL-4) in B-cell lymphoid neoplasms other than chronic lymphocytic leukemia (CLL) such as mantle cell lymphoma and diffuse large B-cell lymphoma. We also found evidence for an induction of the cytosolic levels of DOCK10 by IL-4 in CLL. Finally, we obtained a valid DOCK10 antiserum for immunofluorescence (IF) microscopy that, as an antibody against the hemagglutinin (HA) tag, marked PM ruffles and filopodia in HeLa cells with inducible expression of HA-DOCK10.

## 1. Introduction

Dedicator-of-cytokinesis (DOCK) proteins are the products of a family of 11 genes that act as guanine-nucleotide exchange factors (GEFs) for Rho GTPases [1]. They are characterized by a central CZH-1 domain that interacts with phospholipids of the plasma membrane (PM), and a C-terminal CZH2 domain that harbors the GEF activity. According to sequence homology, DOCK proteins are grouped into four subfamilies, A to D. The DOCK-D or Zizimin subfamily, which comprises DOCK9, DOCK10, and DOCK11, includes an N-terminal pleckstrin homology (PH) domain that interacts with phosphoinositides of the PM [2,3]. Rho GTPases play essential roles in the regulation of actin cytoskeleton dynamics. GEF proteins interact with Rho GTPases and catalyze the exchange of GDP for GTP, thereby activating downstream effectors [4]. DOCK9 and DOCK11 preferentially interact with Cdc42 and induce thin motile PM protrusions, called filopodia, consisting of bundles of unbranched actin filaments [5,6,7]. DOCK10 interacts with both Rac1 and Cdc42 [8,9] and induces both filopodia and membrane ruffles [9]. Ruffles are flat PM protrusions that do not adhere to the substrate and move like waves, and whose cytoskeletal component is a mesh of branched actin filaments.

Immunohistochemical studies performed mainly on cell lines transfected with tagged proteins suggest that DOCK proteins are located in the cytoplasm, and that translocation to the PM is essential for their signaling and functions. For example, DOCK1 transfected in LR73 Chinese hamster ovary (CHO), 293T, Cos-7, or HeLa fibroblast/epithelial cells displayed cytosolic distribution, and upon PDGF or EGF stimulation translocated to the PM [10,11,12]; DOCK2 transfected in HEK293T cells and DOCK3 transfected in HEK293T cells and SW480 colon carcinoma cells displayed cytosolic expression [13,14]; DOCK4 transfected in MDA-MB-231 invasive breast cancer cells was localized at the tips of cortactin-rich PM protrusions [15]; DOCK5 transfected in HeLa, MCF10A non-malignant mammary cells, and MDA-MB-231 cells localized to cytosol, focal adhesions, or lamellipodia [12]; and DOCK6 transfected in HeLa maps at the endoplasmic reticulum [16]. In our inducible model of stable HeLa cell line clones, DOCK10 and DOCK9 localized to ruffles and filopodia [9,17]. However, the lack of valid antibodies (Abs) for immunohistochemistry makes it difficult to study the subcellular localization of endogenous or untagged DOCK proteins. Furthermore, it would be interesting to extend the study to more cell models and apply biochemical studies, since they could lead to interesting observations such as the HeLa S3 cell line, which endogenously expressed higher levels of DOCK1 in the nucleoplasm than in the cytosol [18]. We ourselves have observed that DOCK10 was distributed between cytosol and nucleoplasm, but with higher levels in the latter, in a sample from a patient with chronic lymphocytic leukemia (CLL) [19]. Furthermore, both the cytoplasmic and nuclear levels of DOCK10 were increased by treatment with interleukin-4 (IL-4) in this patient.

In the present study, our first goal was to investigate whether the nuclear localization of DOCK-D proteins is a rare or common occurrence. For this purpose, we fractionated cytosolic and nuclear protein extracts from cell lines and analyzed the expression of DOCK9, DOCK10, and DOCK11. The distribution varied for each cell line, having in some cases similar levels in both compartments and in others a preference for one or another location, with the nuclear location, in conclusion, being quite common. Second, we set out to investigate whether IL-4 induces DOCK10 in other B-cell neoplasms than CLL, and to analyze the cytoplasmic and nuclear distribution in another CLL patient using appropriate indicators of the purity of the cytosolic and nuclear fractions. We found that IL-4 induces the expression of DOCK10 in lymphoid neoplasms such as mantle cell lymphoma and DLBCL, and we confirmed that IL-4 induces cytoplasmic levels of DOCK10 in CLL. Finally, we provide evidence of the first useful DOCK10 antiserum for IF microscopy, which labelled PM ruffles and filopodia in HeLa cells transfected with DOCK10.

## 2. Materials and Methods

### 2.1. Cell Lines

A panel of 11 hematopoietic and epithelial/fibroblast-like cell lines, detailed in Table 1, was used. Hematopoietic cells were grown in RPMI-1640 medium supplemented with 10% fetal calf serum (Biowhittaker, Cambrex, East Rutherford, NJ, USA), 50 U/mL penicillin, 50 U/mL streptomycin, 2.5 μg/mL amphotericin B, and 2 mM L-glutamine. Epithelial/fibroblast-like cells were grown in Dulbecco’s minimum essential medium (DMEM) with the same supplements.

### 2.2. Patients

Whole blood from seven patients with different B-cell neoplasms was obtained including two patients with mantle cell lymphoma (MCL) and one patient from each of these entities: prolymphocytic leukemia (PLL), diffuse large B-cell lymphoma (DLBCL), non-Hodgkin lymphoma (NHL), plasma cell leukemia (PCL), and CLL, diagnosed according to the guidelines of the World Health Organization (WHO) [20]. All the patients had leukocytosis, and none were receiving chemotherapy at the time of sampling. The samples were processed for isolation of B cells using RossetteSep Enrichment Cocktails (StemCell Technologies, Vancouver, Canada). Cell aliquots were cultured for 24 h without or with 10 ng/mL human recombinant IL-4 (BD Pharmingen, BD Biosciences, San Diego, CA, USA) in RPMI-1640 medium with the same supplements described in Section 2.1.

### 2.3. HeLa Stable Cells with Regulatable Expression of DOCK10

Stable clones with regulatable expression of HA-labeled DOCK10 in the cervical cancer epithelial cell line HeLa were previously generated using the tet-off system [9]. Cells were grown in DMEM with supplements plus 1 µg/mL of puromycin, 0.5 mg/mL of G418, and 2 ng/mL of doxycycline (dox). Cells were maintained subconfluent by detachment with trypsin 0.05%-EDTA 0.02% in PBS (EuroClone, Milano, Italy) and routine subculture. Expression of DOCK10 was induced by washing and reseeding the cells in medium lacking dox, or adding dox again to be used as a negative control.

### 2.4. Cytosolic and Nuclear Protein Extraction

The cytosolic and nuclear protein extracts were obtained using the Fermentas ProteoJET Cytoplasmic and Nuclear Protein Extraction Kit (Thermo Fisher Scientific, Waltham, MA, USA). Following the manufacturer instructions, 5 × 10^6^ cells were washed with PBS and the pelleted cells received 200 µL of ice-cold cell lysis buffer. The cells were then vigorously resuspended and incubated on ice for 10 min. Following centrifugation at 500× *g* for 7 min at 4 °C, the supernatants were collected, and the pellets containing the nuclei were reserved. The supernatants were cleaned up by centrifugation at 20,000× *g* for 15 min at 4 °C, and the resulting supernatants, which constitute the cytosolic extracts, were collected. The nuclei-containing pellets, previously reserved, received 1 mL of nuclei washing buffer. The nuclei were then vigorously resuspended, and incubated on ice for 2 min. Following centrifugation at 500× *g* for 7 min at 4 °C, the supernatants were removed, and the pellets received 200 µL of ice-cold nuclei storage buffer. Then, the pellets were resuspended by pipetting up and down from 5 to 10 times, and 20 µL of nuclei lysis buffer was added. Nuclei were incubated with shaking at 1200 rpm for 15 min at 4 °C in a Thermomixer comfort (Eppendorf, Hamburg, Germany), then were centrifuged at 20,000× *g* for 5 min at 4 °C. Finally, the supernatants, which constitute the nuclear extracts, were collected.

### 2.5. Western Blot (WB) Analysis

Immunoreactive proteins were detected as previously described [9]. Briefly, 20 µg of cytosolic and nuclear protein extracts were fractionated in SDS-PAGE gels at 6% for the detection of DOCK9, DOCK10, and DOCK11 and at 10% for the detection of HDAC1 and actin, and electroblotted onto nitrocellulose membranes. Blots were blocked, incubated with primary Abs (Table 2) in TBST with 0.5% skim milk for 2 h, washed, and incubated with horseradish peroxidase (HRP)-conjugated secondary Abs—rabbit, mouse or goat immunoglobulins (Igs) (Table 2)—in TBST with 2.5% skim milk for 1 h. After final washes, immunoreactive proteins were detected using the Amersham ECL Plus Western Blotting Detection Reagents (GE Healthcare) in the Molecular Imager ChemiDoc™ XRS+ with Image Lab software (Bio-Rad Laboratories, Hercules, CA), which provides the tools to measure the intensity of the bands. Abs and their dilutions are listed in Table 2. The primary (1ary) Abs that were obtained from the mouse or rat (Actin, HA) were monoclonal (Mo) and those obtained from the rabbit or goat (DOCK9, DOCK10, DOCK10.1, DOCK11, HDAC1) were polyclonal (Po).

### 2.6. Immunofluorescence (IF) Microscopy

The HeLa clone with regulatable expression of HA-DOCK10 was grown onto 12 mm BioCoat Poly-L-Lysine-coated coverslips (Corning Inc., Corning, NY, USA). Preparations were labelled using the F-actin Visualization Biochem Kit (Cytoskeleton Inc., Denver, CO), following the manufacturer’s instructions with previously described modifications [9]. Briefly, after fixation and permeabilization, coverslips were incubated for 1 h at 4 °C with one of two primary Abs, either a rabbit anti-DOCK10.1 antiserum raised against peptide MAGERTRRFTRSLLRPGQAAEL [21] or a rat anti-HA Ab (Table 2). Following three washes, coverslips were simultaneously labelled with one of two secondary Abs, either anti-rabbit Igs-FITC Igs or anti-rat Igs-Alexa Fluor 488 (Table 2), 100 nM phalloidin-rhodamine (TRITC), and 1 µg/mL DAPI for 30 min at room temperature. After the final washing steps, coverslips were placed onto slides using Dako fluorescent mounting medium. Fluorescence images were acquired by an Eclipse T*i* inverted microscope (Nikon Instruments Inc., Melville, NY, USA) using the NIS Elements software.

## 3. Results

### 3.1. Expression of the DOCK-D Proteins in Cytosolic and Nuclear Fractions of Cell Lines

**Figure 1:**
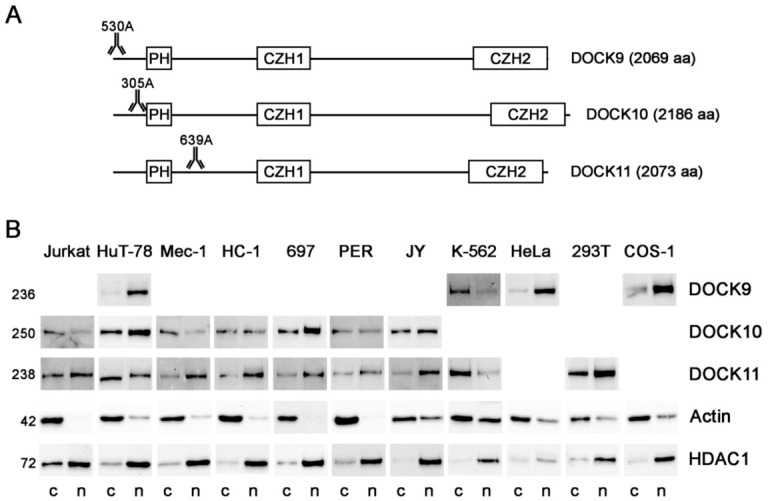
Distribution of DOCK9, DOCK10, and DOCK11 proteins between the cytosol and nucleoplasm of cell lines. (**A**) Diagram of the main domains of the DOCK-D proteins indicating the approximate positions targeted by the Abs used in the present study. 530A, 305A, and 639A are identifiers of the commercial Abs used (short catalog nos., Table 2). (**B**) WB analyses of DOCK9, DOCK10, and DOCK11 expression in the cytoplasmic and nuclear fractions of 11 cell lines using the specific Abs shown in A. The blank spaces correspond to unrealized WB, as no significant levels of the corresponding DOCK protein are expressed in this cell line. WB analyses of actin and HDAC1 expression are shown as indicators of the purity of the cytosolic and nuclear fractions, respectively. The approximate molecular size of each of the proteins studied, in kDa, is shown to the left of the blots. PH, pleckstrin homology; CZH1, CDM-zizimin homology 1; CZH2, CDM-zizimin homology 2; c, cytosol; n, nucleoplasm.

To gain a better understanding of the role of the DOCK-D proteins, their distribution between cytosol and nucleoplasm was investigated in a panel of 11 selected cell lines whose total levels were already known from previous studies [21,22,23]. Specifically, DOCK9 was significantly expressed in four cell lines (HuT-78, K-562, HeLa, and COS-1) [23], DOCK10 in seven cell lines (Jurkat, HuT-78, Mec-1, HC-1, 697, PER, and JY) [21,22], and DOCK11 in nine cell lines (Jurkat, HuT-78, Mec-1, HC-1, 697, PER, JY, K-562, and 293T) [23]. Figure 1A shows a diagram of the main domains of the DOCK-D proteins and the approximate position in which the epitopes recognized by the Abs used are located. DOCK9 was mainly expressed in the nucleoplasm of HuT-78, HeLa, and COS-1, but in the cytosol of K562 (Figure 1B). The expression of DOCK10 was mainly nuclear in HuT-78 and 697, cytoplasmic in Jurkat and Mec-1, and without clear preference in HC-1, PER, and JY. Finally, DOCK11 was expressed mainly in the nucleoplasm of Jurkat, Mec-1, HC-1, 697, PER, JY, and 293T, while its presence was predominant in the cytosol of HuT-78 and K-562. Therefore, the three DOCK-D proteins can be expressed in the cytosol and nucleoplasm, and the balance between both compartments is variable, specific to the cell line.

### 3.2. Induction of Expression of DOCK10 by IL-4 in B-Cell Neoplasms and Distribution between Cytosolic and Nuclear Fractions in a CLL Patient

**Figure 2:**
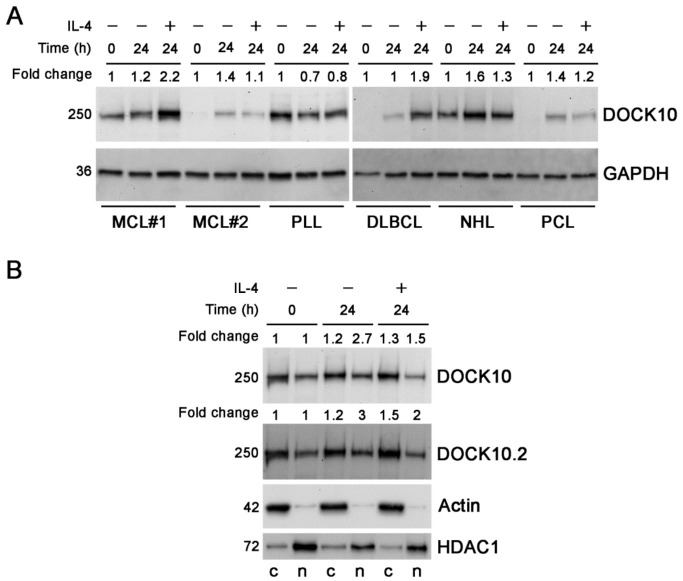
Induction of DOCK10 protein by IL-4 in B-cell lymphoid neoplasms and its distribution between the cytosol and the nucleoplasm in a CLL patient. (**A**) Induction of DOCK10 protein by IL-4 in 6 patients: 2 MCL, 1 PLL, 1 DLBCL, 1 NHL, and 1 PCL. WB analysis of total extracts from each sample before treatment and after 24 h of culture without and with IL-4 using Abs against total DOCK10 (305A) and GAPDH. Fold changes were calculated after normalization with GAPDH in comparison with the T0 sample. (**B**) Distribution of DOCK10 protein between the cytosol and nucleoplasm in a CLL patient. WB analysis of cytoplasmic and nuclear extracts from the sample before treatment and after 24 h of culture without and with IL-4, using Abs against total DOCK10 (305A), the DOCK10.2 isoform, actin, and HDAC1. The fold changes of total DOCK10 and the DOCK10.2 isoform were calculated after normalization by actin for cytosolic levels and by HDAC1 for nuclear levels, in comparison with the respective T0 cytosolic or nuclear fractions. The approximate molecular size of each of the proteins studied, in kDa, is shown to the left of the blots. c, cytosol; n, nucleoplasm.

DOCK10 expression is induced by IL-4 in CLL and normal B cells, but it is not known whether this occurs in other lymphoid neoplasms. To answer this question, we studied some cases of such entities. The expression of DOCK10 was induced by IL-4 in MCL#1 and in the DLBCL patient (Figure 2A). In contrast, DOCK10 was not induced by IL-4 in the PLL patient. Finally, in MCL#2, and in the NHL and PCL patients, slight increases in DOCK10 were observed after culture with IL-4, but these were of similar intensity to those produced by culture without IL-4. In light of these results, it cannot be clearly stated whether or not IL-4 exerted an effect on DOCK10 levels in these patients. 

To obtain more evidence about the distribution of DOCK10 between cytosol and nucleoplasm in CLL, the cytoplasmic and nuclear extracts of a new CLL patient were analyzed, and in this case, the values were normalized with respect to cytoplasmic and nuclear housekeeping proteins, actin and HDAC1, respectively. We found that DOCK10 was also expressed in both cell compartments in this patient, but unlike the case previously studied, the cytoplasmic levels were higher than the nuclear levels (Figure 2B). The cytoplasmic levels of DOCK10 increased due to the effect of IL-4. The nuclear levels of DOCK10 also increased in the culture with IL-4 with respect to the initial sample, but not with respect to the control culture, which does not allow a conclusion to be drawn on whether DOCK10 is induced by IL-4 in the nucleus of the patient. 

### 3.3. Localization of DOCK10 in PM Ruffles and Filopodia in Transfected HeLa Cells Using a DOCK10 Antiserum

**Figure 3:**
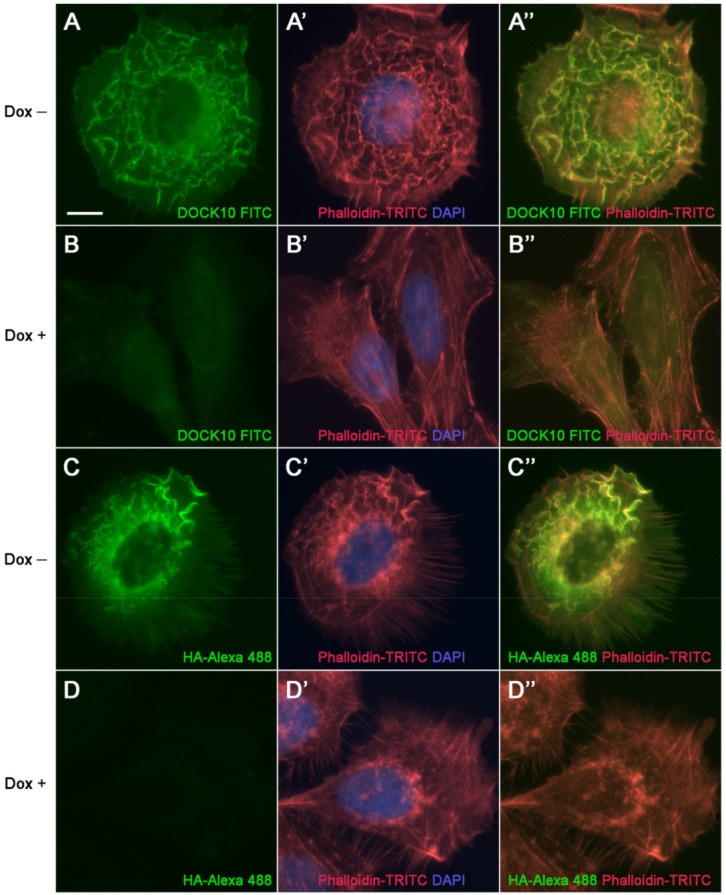
Localization of DOCK10 in PM ruffles in transfected HeLa cells using a DOCK10 antiserum. IF microscopy analysis of HeLa cells with inducible expression of HA-DOCK10, washed free of dox, reseeded without (–) or with (+) dox on poly-L-lysine coating, and cultured for 24 h. Cells were labelled with rabbit anti-DOCK10 antiserum (micrograph series **A**,**B**) or rat anti-HA Ab (micrograph series **C**,**D**), followed by FITC-conjugated swine anti-rabbit Igs (green) or Alexa Fluor 488-conjugated goat anti-rat Igs (green), respectively, phalloidin-TRITC (red), and DAPI (blue). (**A**–**D**) Labelling with FITC- or Alexa Fluor 488-conjugated Igs (**A’**–**D’**) Merge of labelling with phalloidin-TRITC and DAPI (**A”**–**D”**) Merge of FITC- or Alexa Fluor 488-conjugated Igs and phalloidin-TRITC labelling. Micrograph series (**A**,**C**) show DOCK10 localization in PM ruffles, both using the DOCK10 antiserum (**A**) and HA Ab (**C**). Micrograph series B and D represent negative controls for A and C, respectively. Objective magnification: 60×. Scale bar: 10 µm.

To evaluate the usefulness of a DOCK10 antiserum [20] for IF microscopy, we set out to investigate, in parallel to the HA Ab previously used, its performance on HeLa cells with regulatable expression of HA-DOCK10. In cells detached with trypsin-EDTA, washed free of dox, reseeded and cultured for 24 h in the absence of dox, DOCK10 expression levels increased, as did the proportion of cells that presented PM ruffles, filopodia, or both structures simultaneously [9]. In cells developing ruffles, DOCK10 located to them, as shown using both the DOCK10 antiserum (Figure 3A) and the HA Ab (Figure 3C). Visualization of actin (Figure 3A’,C’) showed a high degree of co-localization with DOCK10, with the ruffles turning yellow in the overlapping images (Figure 3A”,C”). Background labeling levels were low, as demonstrated by the observation of cells that followed the same procedure but were cultured for 24 h in the presence of dox, using both the DOCK10 antiserum (Figure 3B,B’,B”) and HA Ab (Figure 3D,D’,D”).

**Figure 4:**
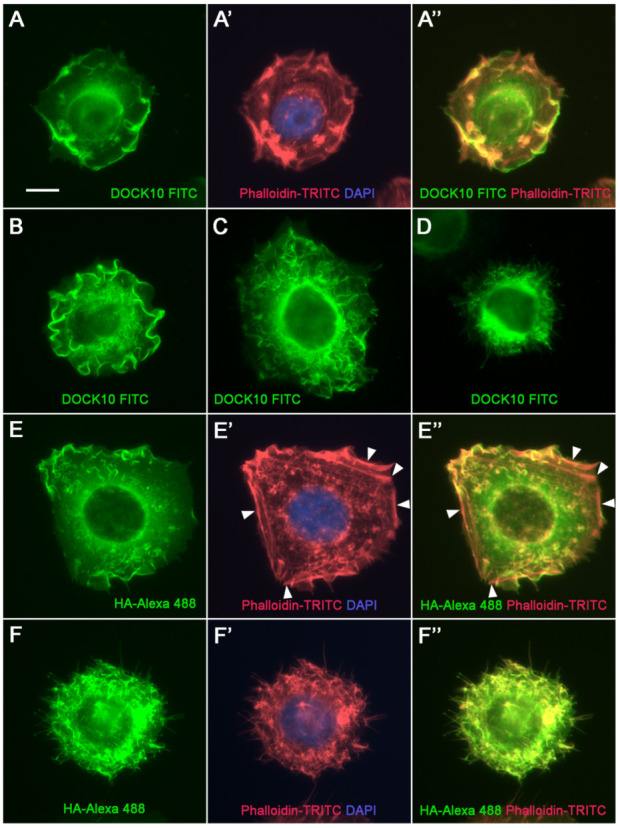
Localization of DOCK10 in PM ruffles and filopodia in transfected HeLa cells using a DOCK10 antiserum. IF microscopy analysis of a HeLa clone with inducible expression of HA-DOCK10, washed free of dox, reseeded without dox on poly-L-lysine coating, and cultured for 24 h. Cells were labelled with rabbit anti-DOCK10 antiserum (micrograph series **A** and micrographs **B**–**D**) or rat anti-HA Ab (micrograph series **E**,**F**), followed by FITC-conjugated swine anti-rabbit Igs (green) or Alexa Fluor 488-conjugated goat anti-rat Igs (green), respectively, phalloidin-TRITC (red), and DAPI (blue). (**A**–**F**) Labelling with FITC- or Alexa Fluor 488-conjugated Igs (**A’**,**E’**,**F’**). Merge of phalloidin-TRITC and DAPI labelling. (**A”**,**E”**,**F”**). Merge of labelling with FITC- or Alexa Fluor 488-conjugated Igs and phalloidin-TRITC. Micrograph series (**A**,**E**), and micrographs (**B**,**C**), illustrate the extensive presence of DOCK10 in the membrane ruffles. Arrowheads in (**E’**,**E”**) indicate peripheral stress fibers. Micrograph (**D**) and micrograph series (**F**) illustrate that in filopodia-rich cells, DOCK10 locates to filopodia. Objective magnification: 60×. Scale bar: 10 µm.

To exemplify the morphological variety of HeLa cells that develop DOCK10-induced ruffles, more micrographs are shown using both the DOCK10 antiserum (Figure 4A–C) and HA Ab (Figure 4E). In cells developing filopodia, DOCK10 located to them, as shown using both the DOCK10 antiserum (Figure 4D) and HA Ab (Figure 4F). Visualization of actin (Figure 4A’,E’,F’) showed co-localization with DOCK10 in ruffles (Figure 4A”,E”) and filopodia (Figure 4F”), but not in other actin structures such as peripheral stress fibers (Figure 4E’,E”, arrowheads). These results demonstrate that the DOCK10 antiserum can be useful for IF microscopy, since it detects the same structures as HA Ab in HeLa cells transfected with HA-DOCK10.

## 4. Discussion

Finding out the subcellular localization of a protein is important because it helps to identify its potential interacting partners and to formulate hypotheses about its potential functions. The guanine exchange function of DOCK proteins occurs through interaction with lipid-bound Rho GTPases on the intracytoplasmic face of the PM. However, there are examples of DOCK proteins that can also be found in non-cytosolic locations such as DOCK1 in the HeLa S3 cell line, which is located in the nucleoplasm. Both in its usual cytosolic location and in its nuclear location, DOCK1 has the ability to interact with ELMO proteins and activate Rac1 [18]. Moreover, a new nuclear function of DOCK1 has recently been discovered in the uterus, consisting of the nuclear import of the autoimmune regulator (AIRE), a mechanism that plays a role in decidualization [24].

In the present paper, to investigate whether the nuclear localization of DOCK-D proteins is rare or frequent, we carried out biochemical studies in cytosolic and nuclear fractions of 11 cell lines, eight of which are hematopoietic and three are fibroblast-like/epithelial, using three commercial Abs directed against total DOCK9, DOCK10, and DOCK11, respectively. We found that nuclear expression of DOCK9, DOCK10, and DOCK11 was more common than expected, since it was observed in most cell lines of the panel. In fact, in the cases of DOCK9 and DOCK11, the predominance of nuclear expression over cytoplasmic expression had a higher incidence (three out of four (75%) and seven out of nine (78%), respectively), although not so much in the case of DOCK10 (two out of seven (29%)). The nuclear function of DOCK-D proteins remains to be investigated.

IL-4 signaling drives proliferation, differentiation, and survival of B lymphocytes from healthy subjects and from diverse B-cell neoplasms such as CLL [25,26,27]. In vitro, IL-4 affects the expression of more than 200 genes and about 10 miRNAs [28,29]. DOCK10 is one of the genes induced by IL-4, especially its DOCK10.2 isoform, which is more expressed in B cells, although the functional impact of this increase is unknown [19,21,22]. In this article, we extend the phenomenon of IL-4 induction of DOCK10 in B lymphocytes beyond normal B lymphocytes and CLL to cases of MCL, a disease entity closely related to CLL and DLBCL. However, it was not seen in one case of B-cell PLL and did not appear to be very significant in other cases including NHL, PCL, and another case of MCL. Therefore, it is not a widespread phenomenon within B neoplasms. Our preliminary observations also suggest that it is extremely rare in B-cell acute lymphoblastic leukemia (B-lymphoblastic leukemia/lymphoma [B-ALL]), suggesting that there could be a relationship with the maturity stage of the diseased B cells (our unpublished observations).

We studied cytoplasmic and nuclear expression of DOCK10 in a CLL sample, before and after treatment with IL-4, and observed a higher expression in the cytosol than in the nucleoplasm, unlike in another case previously studied [19]. Furthermore, in the case cited, IL-4 seemed to increase DOCK10 levels in both compartments, and in the new case of the present work, only an effect of IL-4 on the increase in cytoplasmic levels was clear. These data suggest some variability between CLL in terms of the distribution between the cytosol and the nucleus of DOCK10.

In general, commercially available Abs to DOCK proteins, and others shown in published studies, do not convincingly demonstrate their validity for immunohistochemistry. Either negative controls are not shown or their correct molecular size is not demonstrated by WB [18,30,31,32,33]. In the present paper, we used a DOCK10 antiserum that cleanly detects a band of the appropriate molecular size (249 kDa [21]). In addition, the DOCK10 antiserum labeled ruffles and filopodia in our inducible HeLa clone, the same structures targeted by the Ab against the N-terminal HA tag, providing evidence that our DOCK10 antiserum is useful for IF. We hope that this tool will help investigate the localization of DOCK10 in more cell models as well as tissues and organs, thereby deepening our understanding of the cytoplasmic and nuclear as well as physiological functions of DOCK10.

## Figures and Tables

**Table 1:** Cell lines used in this work.

Cell Line	Cell Lineage	Cell Origin	Source
Jurkat	T lymphocytes	Acute lymphoblastic leukemia	1
HuT-78	T lymphocytes	Sezary syndrome	2
Mec-1	B lymphocytes	Chronic lymphocytic leukemia	3
HC-1	B lymphocytes	Hairy cell leukemia	4
697	B lymphocytes	Acute lymphoblastic leukemia	5
PER	B lymphocytes	Epstein-Barr virus-transformed lymphoblasts	6
JY	B lymphocytes	Epstein-Barr virus-transformed lymphoblasts	7
K-562	Myeloid	Chronic myeloid leukemia	1
HeLa	Epithelial	Cervix carcinoma	8
293T	Uncertain (fibroblastic, epithelial, neuronal…)	Embryonic kidney	1
COS-1	Uncertain (fibroblast-like)	Cercopithecus aethiops kidney	1

1, Given by Christine Chomienne, Saint-Louis Hospital, Paris, France. 2, Given by José Zamorano, San Pedro de Alcántara Hospital, Cáceres, Spain. 3, Given by Francesc Bosch, Clinic Hospital, Barcelona, Spain. 4, Purchased from Leibniz Institute DSMZ-German Collection of Microorganisms and Cell Cultures, Braunschweig, Germany. 5, Given by Rose Ann Padua, Saint-Louis Hospital, Paris, France. 6, Given by Maryline Sasportes, Saint-Louis Hospital, Paris, France. 7, Given by José Antonio Campillo, Department of Immunology, University Clinical Hospital Virgen de la Arrixaca, Murcia, Spain. 8, Given by José Yélamos, Experimental Surgery Unit, University Clinical Hospital Virgen de la Arrixaca, Murcia, Spain.

**Table 2:** Antibodies used in this work.

Protein Target (Clone)	Dilution (Ratio)	Used For	Source	Used As	Catalog No.	Manufacturer
DOCK9	1:2000	WB	Rabbit	1ary	A300-530A	1
DOCK10	1:5000	WB	Rabbit	1ary	A301-305A	1
DOCK11	1:5000	WB	Rabbit	1ary	A301-639A	1
DOCK10.2	1:5000	WB	Rabbit	1ary	Non applicable	2
Actin (C-2)	1:1000	WB	Mouse	1ary	sc-8432	3
HDAC1 (C-19)	1:1000	WB	Goat	1ary	sc-6298	3
Rabbit Igs-HRP	1:2000	WB	Swine	2ary	P0399	4
Mouse Igs-HRP	1:2000	WB	Goat	2ary	P0447	4
Goat Igs-HRP	1:2000	WB	Donkey	2ary	sc-2020	3
DOCK10.1	1:100	IF	Rabbit	1ary	Non applicable	5
HA (3F10)	1:100	IF	Rat	1ary	11 867 423 001	6
Rabbit Igs-FITC	1:100	IF	Swine	2ary	F0054	4
Rat Igs-Alexa Fluor 488	1:100	IF	Goat	2ary	A11006	7

1, Bethyl Laboratories, Montgomery, TX, USA. 2, Ref. [19]. 3, Santa Cruz Biotechnology, Dallas, TX, USA. 4, Dako, Glostrup, Denmark. 5, Ref. [21]. 6, Roche Applied Science, Mannheim, Germany. 7, Invitrogen, Thermo Fisher Scientific.
